# 1- and 2-stage hybrid atrial fibrillation ablation using a biparietal irrigated radiofrequency clamp: A dual-center cohort analysis

**DOI:** 10.1016/j.hroo.2026.03.014

**Published:** 2026-03-19

**Authors:** Luca Aerts, Henri Gruwez, Michiel de Wever, Daniel Ezzat, Samuel Heuts, Justin G.L.M. Luermans, Sevasti-Maria Chaldoupi, Jos G. Maessen, Roberto Lorusso, Herbert Gutermann, Laurent Pison, Bart Maesen

**Affiliations:** 1 Department of Cardiothoracic Surgery, Maastricht University Medical Centre, Maastricht, The Netherlands; 2 Cardiovascular Research Institute Maastricht, Maastricht University, Maastricht, The Netherlands; 3 Department of Cardiology, Ziekenhuis Oost-Limburg, Genk, Belgium; 4 Department of Cardiovascular Sciences, KU Leuven, Leuven, Belgium; 5 Cardiovascular Disease Initiative and Program in Medical and Population Genetics, Broad Institute of MIT and Harvard, Cambridge, Massachusetts; 6 Cardiovascular Research Center and Center for Genomic Medicine, Massachusetts General Hospital, Boston, Massachusetts; 7 Department of Cardiology, Maastricht University Medical Centre, Maastricht, The Netherlands; 8 Hasselt University, Diepenbeek/Hasselt, Belgium

**Keywords:** Atrial fibrillation, Hybrid AF ablation, Thoracoscopic ablation, Catheter ablation, 1-stage procedure, 2-stage procedure

## Abstract

**Background:**

Hybrid thoracoscopic ablation (HA), by using a biparietal irrigated radiofrequency (RF) device for epicardial ablation combined with endocardial techniques, has emerged as a promising treatment for atrial fibrillation (AF). However, findings from the endocardial validation are lacking.

**Objective:**

This study aimed to evaluate findings of endocardial validation after epicardial isolation of the pulmonary veins (PVs) and the left atrial posterior wall (LAPW) during HA, performed either as a 1- or 2-stage procedure using a biparietal irrigated RF clamp.

**Methods:**

In this multicenter study, 57 patients with paroxysmal, persistent, or long-standing persistent AF underwent 1-stage (n = 28) or 2-stage HA (n = 29). During the endocardial part, mapping was performed to assess entrance and exit blocks of the PVs and LAPW and thus evaluate lesion completeness. Periprocedural findings, complications, redo ablations, and clinical outcomes were evaluated.

**Results:**

Patients in the 1-stage group had a longer AF duration (117 vs 33 months; *P* = .009), more long-standing persistent AF (39.3% vs 10.3%; *P* = .032), and fewer previous catheter ablations (39.3% vs 79.3%; *P* = .039). Endocardial validation confirmed isolation of the PVs and LAPW in 96.4% of 1-stage and 86.2% of 2-stage procedures (*P* = .353). Complication rates were low, with no significant differences between the 2 groups. Redo catheter ablations, performed in 6 1-stage patients and 1 2-stage patient, confirmed durable PV and LAPW isolation.

**Conclusion:**

HA using an irrigated RF clamp achieved a high percentage of PV and LAPW isolation during the 1- and 2-stage procedures, confirmed by endocardial validation, with low complication rates and sustained lesion integrity during follow-up.

## Graphical abstract

**Figure:**
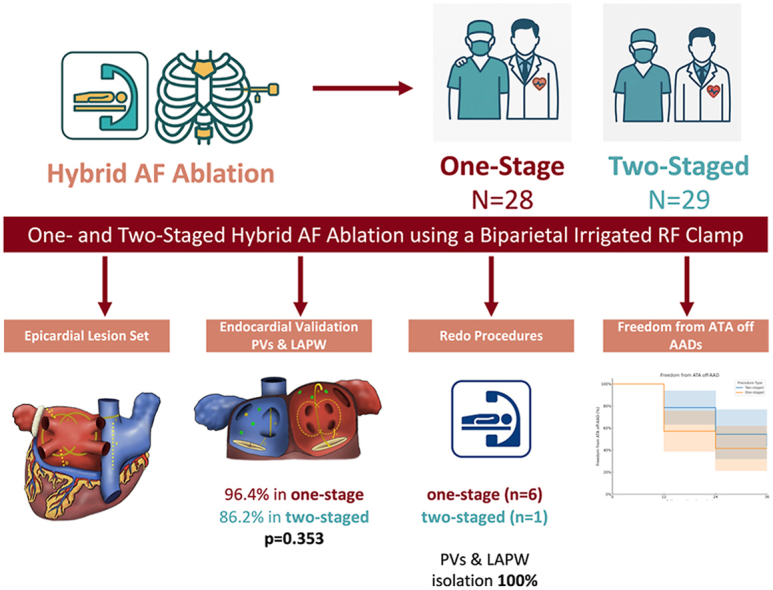

**Key Findings**

▪ Hybrid thoracoscopic ablation using a biparietal irrigated radiofrequency clamp achieved high rates of pulmonary vein and left atrial posterior wall isolation, confirmed by endocardial validation in both 1- and 2-stage procedures.
▪ Endocardial validation demonstrated successful isolation in 96.4% of 1-stage and 86.2% of 2-stage procedures, with no statistically significant difference between the 2 approaches.
▪ Complication rates were low and comparable between 1- and 2-stage hybrid ablation strategies.
▪ Redo catheter ablations showed durable pulmonary vein and posterior wall isolation, indicating sustained lesion integrity during follow-up.

## Introduction

Hybrid atrial fibrillation (AF) ablation integrates 2 complementary techniques. First, a thoracoscopic epicardial ablation is performed to create pulmonary vein (PV) and left atrial posterior wall (LAPW) isolation and address the left atrial appendage (LAA). This is followed by a transvenous endocardial procedure, which enables 3-dimensional electroanatomic mapping of the left and right atria. The endocardial step allows precise localization of residual conduction gaps and touch-up radiofrequency (RF) ablation and induction, mapping, and ablation of other atrial arrhythmias, such as mitral isthmus or cavotricuspid isthmus (CTI) dependent flutter, when necessary.^1^

Despite its clinical relevance, detailed electrophysiological (EP) data on lesion validation during the endocardial component of hybrid AF ablation remains scarce. Moreover, although clinical protocols differ between 1- and 2-stage procedures, the impact of these strategies on intraprocedural findings has not been studied before. Therefore, this study focused on endocardial findings of epicardial lesions created with a biparietal irrigated RF clamp in both 1- and 2-stage hybrid procedures.

## Methods

### Study design

The current study was a dual-center retrospective analysis. The study adhered to the principles outlined in the Declaration of Helsinki and was conducted in accordance with applicable ethical and regulatory standards for human clinical research. The study was designed, conducted, and reported in compliance with internationally accepted guidelines for clinical investigations. After local ethical approval in both centers, the study was registered at Maastricht University Medical Center (MUMC+) (registration number: METC 2022-3561). Data sharing, in anonymized form, was conducted in accordance with the permissions granted by the ethics committees and board committees of the Ziekenhuis Oost-Limburg (ZOL). An informed consent was waived by the institutional review board because the data were pseudo-anonymized and, given the non–Medical Research Involving Human Subjects Act and retrospective nature of the study, presumed consent was considered applicable.

### Study population

This study included consecutive patients with symptomatic paroxysmal AF (PAF), persistent AF (PersAF), and long-standing PersAF (LSPAF), who underwent 1-stage hybrid AF ablation at MUMC+, The Netherlands, and 2-stage hybrid AF ablation at ZOL, Genk, Belgium, using a bipolar biparietal irrigated RF clamp between 2019 and 2023. Only patients with a minimum follow-up of 12 months were included in the analysis.

### Hybrid AF ablation: The epicardial procedure

In this study, thoracoscopic epicardial lesions were created on the beating heart using a biparietal clamp. By using irrigated RF energy, this clamp creates 2 U-shaped lesions by inserting the clamp from 2 sides via a bilateral thoracoscopic approach.^2^, ^3^, ^4^ At the start of the procedure, a transesophageal echocardiogram was performed to confirm the absence of intracardiac thrombi, specifically in the LAA. The procedure was conducted under general anesthesia with double-lumen endotracheal intubation. 3 incisions were made on the left thoracic side: a 5-mm camera port was placed in the fifth intercostal space (midaxillary line), and 2 5-mm working ports were placed in the third and seventh intercostal spaces (anterior axillary line) as previously described.^1^ The pericardium was opened posterior to the phrenic nerve, and the pericardial reflections were opened via the oblique and transverse sinuses using blunt dissection. 2 guides were placed in the sinuses to facilitate the placement of the clamp. The same incisions were made on the right thoracic side, followed by opening the pericardium anterior to the phrenic nerve. The 2 guiding glides from the left sides were retrieved and attached to the 2 jaws of the irrigated RF clamp. The clamp was then positioned around the left and right PVs to create a U-shaped lesion on both the right and left sides, isolating the PVs together with the LAPW. The superior vena cava (SVC) was also ablated epicardially using the same device. The epicardial procedure was completed by transesophageal echocardiogram–guided occlusion of the LAA by use of an epicardial clipping device (Atriclip Pro 2, AtriCure Inc, Cincinnati, OH). In the 2-stage procedures, epicardial testing was performed, confirming exit and entrance blocks of the PVs and the LAPW.

### Hybrid AF ablation: The endocardial procedure

The epicardial ablation was followed by the endocardial procedure. Using a femoral venous approach, a coronary sinus catheter (Medtronic) and a mapping catheter (Lasso, Pentaray, or Octaray, Biosense Webster) were placed under fluoroscopy. After transseptal puncture, an activated clotting time of >300 seconds was maintained. In case of sustained AF, an electrical cardioversion was performed to restore sinus rhythm (SR).

A voltage map of the PVs, LAPW, and the SVC was acquired. Complete isolation (entrance and exit block) of all PVs and the LAPW was checked. Incomplete lesions were addressed with touch-up ablation using a 3.5-mm irrigated-tip RF catheter (SmartTouch, Biosense Webster): posterior 25–30 W 400 ablation index and anterior 30–40 W 550 ablation index in the MUMC+.

In ZOL, when SR was restored, an additional biatrial CARTO finder map was created to identify focal or reentry sources, which were selectively ablated. A CTI line was performed in both 1- and 2-stage endocardial procedures as standard workup. A lateral or anterior mitral isthmus line was performed in case of low-voltage zones in the anterior left atrial wall or based on (a history of) mitral isthmus–dependent flutter. Bidirectional block of the additional ablation lines was confirmed. In the 2-stage procedures, endocardial validation and any necessary touch-up or additional ablations were performed 3 months after the epicardial ablation.

### Objectives

The primary objective of the study was to evaluate endocardial confirmation of epicardial isolation of the PVs and the LAPW during hybrid ablation in both 1- and 2-stage procedures. The secondary objectives were other endocardial findings during endocardial validation and endocardial findings during redo endocardial procedures owing to recurrences: to evaluate the durability (or reconnection) of epicardial isolation of PVs and LAPW and to identify EP mechanisms responsible for atrial tachyarrhythmia (ATA) recurrences. Furthermore, freedom from ATA lasting ≥30 seconds, documented by 12-lead electrocardiography, Holter monitoring, or an implantable loop recorder (ILR) beyond the 3-month blanking period, with follow-up assessments at 12 months and, when available, at 24 and 36 months, was assessed. A reveal (ILR) was only used in ZOL, whereas intermittent monitoring using a Holter was performed in the MUMC+. This was evaluated both with and without the use of antiarrhythmic drugs (AADs). In addition, 30-day periprocedural morbidity and mortality were assessed.

### Statistical analysis

Categorical variables were presented as numbers and percentages (%), and groups were compared using the χ^2^ test. In case of a cell count of <5, Fisher’s exact test was used, as appropriate. The distribution of continuous variables was assessed by visual inspection of histograms and statistically by use of the Shapiro-Wilk test, for which *P* < .05 denoted significant deviation from a normal distribution. Depending on the distribution, these variables were presented as either means and standard deviations or medians and interquartile ranges. In addition, the variables were compared using the Student *t* test (normal distributions) and the Mann-Whitney U test (non-normal distributions). The secondary endpoints (freedom from ATA allowing and off AAD) were presented in Kaplan-Meier curves. These outcome data were compared using the log-rank test and quantified using an unadjusted Cox-regression model, expressed as hazard ratios (HRs) with corresponding 95% confidence intervals (CIs). In addition, a multivariable Cox-regression model was fitted to evaluate the potential mediating effects of AF type (PAF) and previous catheter ablation (CA), expressed in adjusted HRs with 95% CIs. For all analyses, *P* < .05 indicated the presence of a statistically significant difference. All analyses were performed in SPSS (Mac, version 29, IBM Corp, Armonk, NY).

## Results

### Overall patient characteristics

A total of 57 patients were included in the analysis, with 28 undergoing a 1-stage and 29 undergoing a 2-stage hybrid AF ablation. Baseline characteristics are presented in Table 1. The mean patient age was 63 ± 7 years, and the cohort was predominantly male, with females comprising only 21.1% of the study population. Most patients (64.9%) had non-PAF, whereas 35.1% presented with PAF. The median duration of clinical AF history was 54 months (interquartile range 18–120), and 66.7% of patients had previously undergone CA.

**Table 1:** Baseline patient characteristics

Variable	Overall cohort (N = 57)	1-stage (n = 28)	2-stage (n = 29)	*P* value
Patient characteristics				
Preoperative rhythm history, n (%)				.032
Paroxysmal AF	20 (35.1)	9 (32.1)	11 (37.9)	
Persistent AF	23 (40.4)	8 (28.6)	15 (51.7)	
Long-standing persistent AF	14 (24.6)	11 (39.3)	3 (10.3)	
Previous catheter ablation, n (%)	38 (66.7)	15 (53.6)	23 (79.3)	.039
1 previous catheter ablation	11 (28.9)	3 (20)	8 (34.8)	
2 previous catheter ablation	23 (60.5)	10 (66.7)	13 (56.5)	
3 previous catheter ablation	4 (10.5)	2 (13.3)	2 (8.7)	
Number of previous catheter ablations, median [IQR]	2 [2–2]	2 [2–2]	2 [2–2]	.408
AF duration, mo, median [IQR]∗	54 [18–120]	117 [55–184]	33 [12–57]	.009
Age, y, mean (SD)	63 (7)	65 (8)	61 (6)	.557
Female sex, n (%)	12 (21.1)	4 (14.3)	8 (27.6)	.331
BMI, kg/m^2^, mean (SD)	28.2 (3.6)	26.9 (3.5)	29.8 (3.6)	.058
Hypertension, n (%)	33 (57.9)	19 (67.9)	14 (48.3)	.134
Diabetes mellitus, n (%)	5 (8.8)	4 (14.3)	1 (3.4)	.194
Peripheral vascular disease, n (%)	2 (3.5)	1 (3.6)	1 (3.4)	1.000
Myocardial infarction, n (%)	4 (7.0)	4 (14.3)	0	.052
Stroke, n (%)	5 (8.8)	3 (10.7)	2 (6.9)	.670
COPD, n (%)	7 (12.3)	4 (14.3)	3 (10.3)	.706
Congestive heart failure, n (%)	5 (8.8)	4 (14.3)	1 (3.4)	.194
Sleep apnea, n (%)	8 (14.0)	6 (21.4)	2 (6.9)	.144
Smoking history, n (%)				.654
Current	18 (31.6)	10 (35.7)	8 (27.6)	
Previous	9 (15.8)	5 (17.9)	4 (13.8)	
CHA_2_DS_2_-VASc score, median [IQR]	1 [1–3]	1 [1–3]	1 [0–2]	.094
Preoperative medication				
Antiarrhythmic drugs, n (%)†				.669
No antiarrhythmic drugs	33 (57.9)	17 (60.7)	16 (57.1)	
Flecainide	6 (10.5)	3 (10.7)	3 (10.7)	
Procainamide	0	0	0	
Sotalol	4 (7.0)	3 (10.7)	1 (3.6)	
Amiodarone	12 (21.1)	5 (17.9)	7 (25.0)	
Other	0	0	0	
Beta blocker, n (%)	37 (64.9)	17 (60.7)	20 (69.0)	.514
Digoxin, n (%)	8 (14.0)	7 (25.0)	1 (3.4)	.025
Oral anticoagulation, n (%)	49 (86.0)	24 (85.7)	25 (86.2)	1.000
Preoperative transthoracic echocardiography				
LVEF, %, median [IQR]	65% [55–65]	55 [45–58]	65 [65–65]	<.001
LAD, mm, mean (SD)	47 (8)	47 (8)		
LAV, mL, median [IQR]	95 (73–111)	95 (73–111)		
LAVI, mL/m^2^, median [IQR]	45 (33–56)	45 (33–56)		
Mitral valve regurgitation, n (%)‡	4 (7.0)	3 (10.3)	1 (3.8)	.421
Tricuspid valve insufficiency, n (%)§	3 (5.3)	2 (7.1)	1 (3.8)	.241

AF = atrial fibrillation; BMI = body mass index; COPD = chronic obstructive pulmonary disease; IQR = interquartile range; LAD = left atrial diameter; LAV = left atrial volume; LAVI = left atrial volume index; LVEF = left ventricular ejection fraction; PAF = paroxysmal atrial fibrillation; SD = standard deviation.

∗ 4 patients with missing AF duration data in the 2-stage group and 2 patients with missing AF duration in the 1-stage group.

† 1 patient with missing antiarrhythmic drug data in the 2-stage group.

‡ Defined as moderate or more severe regurgitation.

§ Defined as moderate or more severe regurgitation.

### Patient characteristics by procedure

When comparing the 1- and 2-stage hybrid ablation groups, significant differences in patient characteristics were observed. Rhythm history differed significantly (*P* = .032), with a higher proportion of LSPAF in the 1-stage group (39.3% vs 10.3%; *P* = .032) and a greater prevalence of PersAF in the 2-stage group (51.7% vs 28.6%; *P* = .032). Previous CA was more common in the 2-stage group (79.3% vs 53.6%; *P* = .039). Notably, preprocedural AF duration was significantly longer in the 1-stage group than the 2-stage group (117 months [55–184] vs 33 months [12–57]; *P* = .009).

### Epicardial procedural characteristics

Patients in the 2-stage group were significantly more likely to be in SR at the start of the epicardial procedure than those in the 1-stage group (58.6% vs 26.9%; *P* = .010) (Table 2). In all patients (100%), both PVs and posterior box lesions were successfully ablated thoracoscopically. Ablation of the SVC was performed in 96.4% of the patients in the 1-stage group and 96.6% in the 2-stage group (*P* = 1.000). 1 patient in the 1-stage group underwent only the epicardial procedure owing to suspected bleeding, with the endocardial part deferred to a later stage. The LAA was managed in 96.4% of patients in the 1-stage group (n = 1 not performed owing to absence of the LAA) and in 100% of those in the 2-stage group (*P* = .491).

**Table 2:** Procedural characteristics

Variable	Overall cohort (N = 57)	1-stage (n = 28)	2-stage (n = 29)	*P* value
SR at start of procedure, n (%)	24 (42.1)	7 (26.9)	17 (58.6)	.010
Epicardial lesions, n (%)				
PVs + box lesion	57 (100)	28 (100)	29 (100)	1.000
SVC	55 (96.5)	27 (96.4)	28 (96.6)	1.000
Mitral isthmus line	1 (1.8)	1 (3.6)	0	.491
Intercaval line	1 (1.8)	1 (3.6)	0	.491
LAA–LSPV	1 (1.8)	1 (3.6)	0	.491
LAA management	56 (98.2)	27 (96.4)	29 (100)	.491
Additional endocardial lines, n (%)				
CTI	28 (93.0)	28 (100)	25 (86.2)	.112
Mitral isthmus line	11 (19.3)	9 (32.1)	2 (6.9)	.021
Extra ablation of the LA	3 (5.3)	0	3 (10.3)	.237
Extra ablation of the RA	3 (5.3)	0	3 (10.3)	.237
RAA	2 (3.5)	0	2 (6.9)	.491
Intercaval line	1 (1.8)	1 (3.6)	0	.491
SVC	2 (3.5)	2 (7.1)	0	.237

CTI = cavotricuspid isthmus line; LA = left atrium; LAA = left atrial appendage; LSPV = left superior pulmonary vein; PV = pulmonary vein; RA = right atrium; RAA = right atrial appendage; SR = sinus rhythm; SVC = superior vena cava.

**Figure 1:**
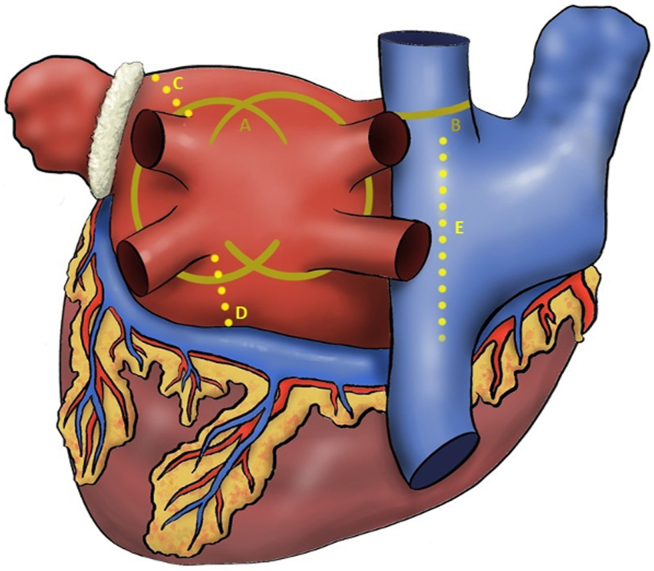
Overview of all potential epicardial lesions: (**A**) pulmonary veins and box lesion, (**B**) superior caval vein lesion, (C) lesion from the left superior pulmonary vein to the left atrial appendage, (**D**) mitral isthmus line, and (**E**) intercaval line.

Additional epicardial ablations were performed in 3 patients (10.7%) in the 1-stage group, whereas no additional epicardial lines were performed in the 2-stage group (*P* = .237). In 1 patient (3.6%), a mitral isthmus line was completed epicardially owing to persistent atrial tachycardia (AT) with previous endocardial AT ablations. In another patient (3.6%), a line from the LAA to the left superior PV was created, based on the location of a previously ablated AT originating from the base of the LAA. In a third patient, an intercaval line was added owing to marked right atrial dilatation. All potential epicardial lesion sets are presented in Figure 1.

### Endocardial procedural characteristics

**Figure 2:**
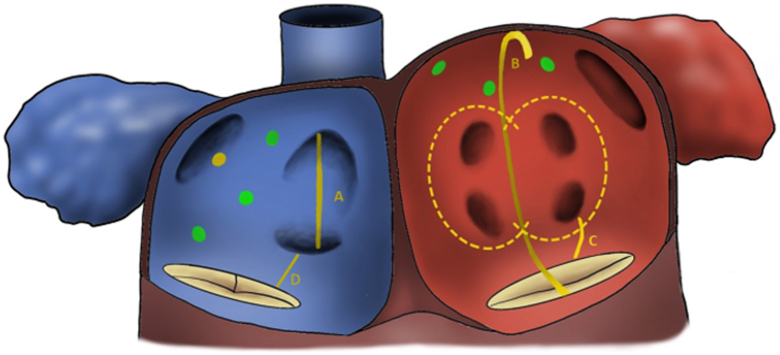
Overview of all endocardial lesions: (**A**) intercaval line, (**B**) anterior mitral isthmus line, (**C**) lateral mitral isthmus line, and (**D**) cavotricuspid isthmus line. *Green dots* indicate additional ablations in the left and right atria. The *yellow dot* denotes additional applications at the base of the right atrial appendage. Lesions depicted with the *dashed yellow line* represent epicardial applications.

During endocardial validation, there were no significant differences in exit and entrance blocks of the box lesion between patients in the 1-stage (27 patients, 96.4%) and the 2-stage group (25 patients; 86.2%) (*P* = .353). In the 1-stage group, 1 patient (3.6%) showed no entrance or exit block within the box lesion, necessitating endocardial reisolation of both the left and right PVs and the floor line. In the 2-stage group, 3 patients (10.3%) demonstrated incomplete box isolation. Among these, 2 underwent endocardial reisolation of both the roof and floor lines, whereas 1 required reisolation of the floor line. For patients who underwent an epicardial SVC lesion procedure, isolation was confirmed in 27 patients (100%) in the 1-stage group and 28 patients (100%) in the 2-stage group (*P* = 1.000). A CTI line was performed in 28 patients (100%) in the 1-stage group and in 25 patients (86.2%) in the 2-stage group. In addition, a mitral isthmus line was performed in 9 patients (32.1%) in the 1-stage group and in 2 patients (6.9%) in the 2-stage group. In 2 patients, the SVC ablation was completed endocardially; in 1 of these patients, this was deferred to the second-stage endocardial procedure owing to bleeding. In 1 patient, the SVC line was approximated. All additional endocardial lines are presented in Table 2, whereas all potential endocardial lesions—each completed with confirmed bidirectional block—are presented in Figure 2.

### Postoperative course and early outcomes

The postoperative course and early outcomes are presented in Table 3. Overall, most outcomes did not differ significantly between patients undergoing single-stage vs 2-stage hybrid AF ablation. However, a significant difference was observed in the duration of intensive care unit (ICU) admission, with patients in the 2-stage group having a longer ICU stay (2 days vs 1 day; *P* < .001) and total hospital stay (6 days vs 5 days; *P* < .001).

**Table 3:** Postoperative course and early outcomes

Variable	Overall cohort (N = 57)	1-stage (n = 28)	2-stage (n = 29)	*P* value
ICU admission, d, median [IQR]	2 [1–2]	1 [1–1]	2 [2–2]	<.001
Hospital admission, d, median [IQR]	5 [4–7]	4 [4–5]	6 [6–8]∗	<.001
Rhythm during admission, n (%)				.467
Sinus rhythm	46 (80.7)	22 (78.6)	24 (82.8)	
AF	8 (14.0)	5 (17.9)	3 (10.3)	
AF + atrial flutter	1 (3.5)	0	1 (3.4)	
Junctional rhythm	1 (3.5)	0	1 (3.4)	
Junctional rhythm + AF + atrial flutter	1 (3.5)	1 (3.6)	0	
In-hospital mortality (%)	0	0	0	NA
Postoperative complications, n (%)				
Conversion to sternotomy	1 (1.8)	0	1 (3.4)	1.000
Pneumothorax requiring drainage	0	0	0	NA
Hemothorax/pleural effusion requiring drainage	2 (3.5)	2 (7.1)	0	.237
Respiratory failure	0	0	0	NA
Pneumonia	1 (1.8)	0	1 (3.4)	1.000
Pericarditis	2 (3.5)	0	2 (6.9)	.491
Stroke	0	0	0	NA
Permanent PM implantation	0	0	0	NA
Phrenic nerve palsy	0	0	0	NA
Ventricular fibrillation	0	0	0	NA
Myocardial infarction	1 (1.8)	0	1 (3.4)	1.000
Acute kidney injury	1 (1.8)	1 (3.6)	0	.491

AF = atrial fibrillation; ICU = intensive care unit; IQR = interquartile range; NA = not available; PM = pacemaker.

∗ This is the sum of 2 hospitalizations (epicardial and endocardial procedures).

Postoperative complications did not differ significantly between groups, and no mortality was reported in the total cohort. 1 patient in the 1-stage group required conversion to sternotomy owing to uncontrollable bleeding of 1 of the PVs. The endocardial part was performed in a second procedure. Overall, complication rates remained low, with 2 patients developing hemothorax requiring drainage (3.5%), 1 patient having pneumonia (1.8%), and 1 patient having a periprocedural myocardial infarction (1.8%). There were no postoperative pacemaker implantations.

### Freedom from ATA

**Figure 3:**
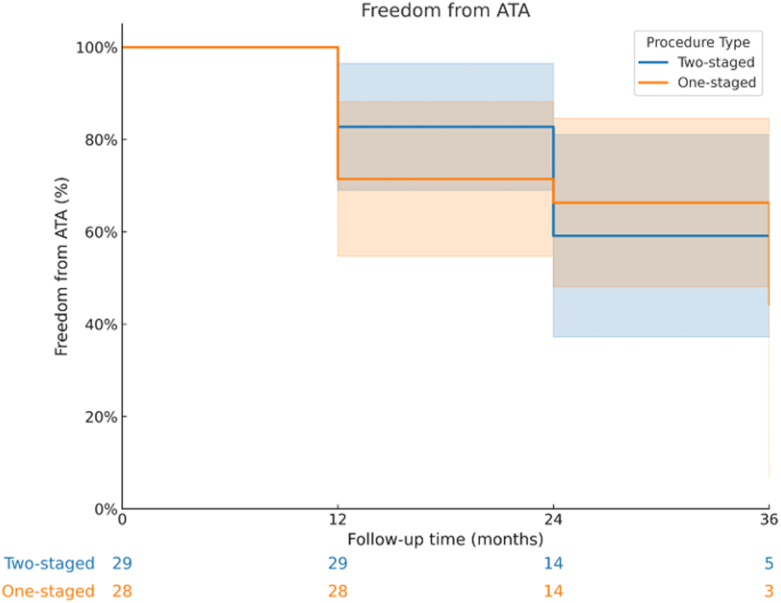
Kaplan-Meier curves for freedom from ATA for all patients allowing antiarrhythmic drug in 1- and 2-stage hybrid ablation. ATA = atrial tachyarrhythmia.

Overall freedom from ATA allowing AAD in the 1-stage group was 71.4%, 66.3%, and 44.2% at 1, 2, and 3 years of follow-up, respectively, and 82.8%, 59.1%, and 59.1% in the 2-stage group, without a significant difference between groups (log-rank *P* = .734; HR 0.86; 95% CI 0.35–2.11) (Figure 3). Notably, we did not observe significant mediating effects of previous CA or type of AF on the outcome of freedom from ATA (HR staging 0.65; 95% CI 0.22–1.87; HR before CA 1.59; 95% CI 0.52–4.88; HR PAF 1.63; 95% CI 0.54–4.94).

**Figure 4:**
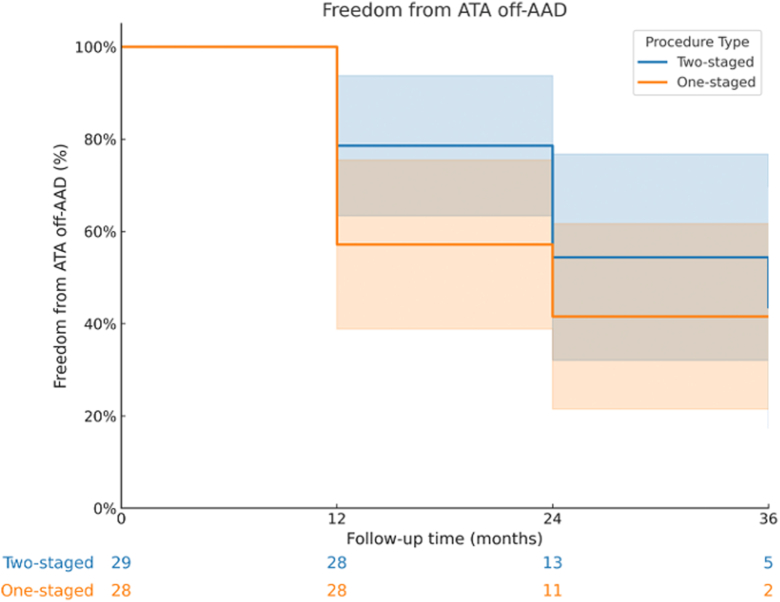
Kaplan-Meier curves for freedom from ATA for all patients off AAD in 1- and 2-stage hybrid ablation. AAD = antiarrhythmic drug; ATA = atrial tachyarrhythmia.

Overall freedom from ATA off AAD in the 1-stage group was 57.1%, 41.6%, and 41.6% at 1, 2, and 3 years of follow-up, respectively, and 78.6%, 54.4%, and 29.0% in the 2-stage group, without a significant difference between groups (log-rank *P* = .383; HR 0.71; 95% CI 0.33–1.53) (Figure 4). Again, we did not observe significant mediating effects of previous CA or type of AF for freedom from ATA off AAD (HR staging 0.66; 95% CI 0.27–1.58; HR before CA 1.69; 95% CI 0.68–4.24; HR PAF 1.18; 95% CI 0.44–3.15).

### Redo catheter procedures

In the 1-stage hybrid AF ablation cohort, redo CA was performed in 6 patients (21.4%) at a mean interval of 15.4 ± 6.2 months after the procedure (Table 4). The indications for redo ablation included atrial flutter (n = 3), PAF (n = 2), and PersAF (n = 1). In the 2-stage group, 2 patients (6.9%) required a redo catheter procedure; however, 1 patient with recurrence of PAF at 16 months of follow-up declined further treatment and was lost to follow-up (Table 5). In this group, 1 patient (3.4%) had a redo procedure at 8 months after the procedure owing to atrial flutter.

**Table 4:** Redo procedures in the 1-stage procedure

Time to recurrence (mo)	Recurrence rhythm	Time to redo procedure (mo)	Endocardial procedure	Follow-up
4	Atrial flutter	6	Persistent PVs, box isolation • Redo anterior MI + CTI	12 mo: AT/AF 24 mo: AT/AF 36 mo: AT
4	PAF	8	Persistent PVs, box, SVC, CTI isolation • Ablation at the base of the LAA • Antralization SVC	12 mo: SR 24 mo: SR 36 mo: SR
4	PAF	6	Persistent PVs, box, SVC, CTI isolation • Antralization anterior wall RPVs	12 mo: AF 24 mo: AF
8	Atrial flutter	14	Persistent PVs, box, SVC, CTI, lateral MI isolation • Ablation under lateral MI • Ablation at the base of the LAA	24 mo: SR
10	Atrial flutter	18	Persistent PVs, box lesion • Redo CTI • Anterior MI	24 mo: SR 36 mo: AT 48 mo: SR 50 mo: SR
11	PersAF	23	Persistent PVs, box isolation • Redo ablation of the SVC • Anterior MI • Ablation of low-voltage zones (anterior + posterior)	24 mo: AF 36 mo: SR 48 mo: AF 50 mo: AF

AF = atrial fibrillation; AT = atrial tachycardia; CTI = cavotricuspid isthmus line; LAA = left atrial appendage; MI = mitral isthmus; PAF = paroxysmal atrial fibrillation; PersAF = persistent atrial fibrillation; PV = pulmonary vein; RPV = right pulmonary vein; SR = sinus rhythm; SVC = superior vena cava.

**Table 5:** Redo procedures in the 2-stage procedure

Time to recurrence (mo)	Recurrence rhythm	Time to Redo Procedure (mo)	Endocardial procedure	Follow-up
5	Atrial flutter	8	Persistent PVs, box isolation • Ablation of perimetral flutter • Ablation of the microreentry circuit at the base of the LAA	12 mo: AF
16	PAF	-	Planned ablation (lost to follow-up)	-

AF = atrial fibrillation; LAA = left atrial appendage; PAF = paroxysmal atrial fibrillation; PV = pulmonary vein.

Importantly, in all patients undergoing a redo procedure in both groups, the PVs and the associated LAPW showed persistent electrical isolation upon endocardial mapping. No reconnection of the hybrid lesion set was observed. Consequently, additional endocardial ablations or antralization of the existing lesions was performed as the intervention. These redo interventions were substrate guided and targeted non-PV arrhythmogenic regions, most commonly located in the anterior left atrium, mitral isthmus, and LAA region, and included anterior line completion, mitral isthmus ablation, LAA isolation, focal AT ablation, and SVC isolation when indicated. Unfortunately, most patients (57.1%) experienced recurrences during follow-up.

## Discussion

This study presents the findings of 1- and 2-stage hybrid AF ablation, using a biparietal irrigated RF clamp. Our results demonstrate that both approaches, 1- and 2-stage procedures, result in a high percentage of PV and LAPW isolation during endocardial validation, with no observed statistically significant differences in lesion completeness between the 2 groups. Furthermore, hybrid AF ablation is a safe procedure for AF treatment, with a low rate of periprocedural complications. In patients who underwent redo procedures for recurrent arrhythmias in their follow-up, the PVs and LAPW were found to be electrically isolated in all cases, showing durable lesions over time in these patients. Despite this durable isolation of the PVs and LAPW, most patients still experienced arrhythmia recurrence, indicating that factors beyond PVs or posterior wall reconnection—such as non–box-related triggers or atrial substrate progression—may contribute to long-term arrhythmia burden.

### Lesion completeness

A key advantage of the hybrid AF ablation approach lies in the integration of thoracoscopic epicardial ablation with catheter-based endocardial mapping and ablation. This combined strategy allows targeted treatment of both triggers and substrate while enabling lesion validation. During the endocardial procedure, electrophysiologists can apply additional, substrate-specific ablations—such as CTI lines, mitral isthmus lines, and focal ablations that are not feasible epicardially. Moreover, EP mapping during the endocardial step primarily serves to validate lesion completeness and transmurality of previous ablation lesions.^1^^,^^5^

EP confirmation of lesion transmurality remains crucial, given that incomplete epicardial lines can lead to highly symptomatic ATA.^6^ Vroomen et al^7^ previously demonstrated that epicardial and endocardial validation may correlate well, although 15% of right superior PV isolation were not confirmed epicardially, emphasizing the added value of EP mapping. Similarly, the HARTCAP-AF study^8^ reported epicardial conduction gaps in 42% of patients, necessitating endocardial touch-up. Notably, in the HARTCAP-AF study, a nonirrigated clamp was used for PV isolation, whereas a separate unidirectional device was used for the roof and floor lines, potentially increasing the likelihood of conduction gaps at the line junctions. In contrast, the irrigated bipolar clamp used in our study creates a continuous, U-shaped lesion from the left and the right side, encompassing the PV antra, roof, and floor, thereby reducing the likelihood of lesion discontinuity and minimizing the need for endocardial touch-up. Although the present study focused on the irrigated bipolar clamp technology, the favorable lesion durability likely reflects a combination of both the irrigated energy delivery and the all-clamp box lesion strategy. The importance of a clamp-based continuous box approach—also described with nonirrigated bipolar clamps in thoracoscopic and surgical settings—suggests that lesion set design and energy modulation together determine transmurality and continuity.^9^^,^^10^

### Efficacy and safety outcomes

In our cohort, long-term freedom from ATA off AADs in the 1-stage group was lower than previously reported in some studies, with 57.1%, 41.6%, and 41.6% at 12, 24, and 36 months, respectively. For example, the HARTCAP study^8^ reported 89% freedom from ATA at 12 months off AADs. However, several important differences between the study populations should be considered when interpreting these findings. Patients in the current 1-stage group were in a more advanced disease stage: 39.3% had LSPAF, 53.6% had undergone previous CA, the mean AF duration exceeded 9 years, and the average left atrial volume index was 45 mL/m^2^. In contrast, the HARTCAP study exclusively included patients with PersAF, with a substantially shorter mean AF duration of 22 months, and considered entirely of ablation-naïve patients. These factors are known to negatively affect rhythm outcomes after ablation and may partly explain the observed differences in efficacy.^6^ Although the HARTCAP-AF population consisted exclusively of patients with PersAF—similar to our 2-stage cohort in terms of AF type—all were ablation naïve, whereas 80% of patients in our 2-stage group had undergone previous CA, potentially reflecting a more advanced atrial substrate (eg, greater fibrosis or atrial enlargement) despite shorter AF duration.

Compared with the study of Maesen et al,^11^ which reported arrhythmia-free survival rates of 83%, 80%, and 80% at 1, 2, and 3 years in patients with PAF and 82%, 79%, and 79% in patients without PAF after a 1-stage hybrid AF ablation without class 1 or 3 AADs and without redo CA, differences in patient characteristics become apparent.^11^ The population described by Maesen et al^11^ seems to be more comparable with our cohort, given that it included patients with PAF, PersAF, and LSPAF, with a mean AF history of 59 months, and 35% had undergone previous CA. Nevertheless, the outcomes in our study were less favorable, which is likely attributable to an even more advanced and diseased patient population, as reflected by longer AF duration and a higher burden of previous interventions.

Compared with the CEASE-AF trial—a multicenter randomized controlled trial including only patients without PAF treated with either CA or a 2-stage hybrid approach—the present results show a mixed pattern over time.^12^ In the CEASE-AF trial, freedom from ATA was 74.7% at 12 months allowing AADs, 63.2% off AADs, and 61.1% at 36 months. In comparison, our 2-stage cohort demonstrated a higher freedom from ATA off AADs at 12 months (78.6%), but a substantially lower rate at 36 months (29%). This decline suggests limited long-term durability of rhythm control in this advanced AF population and underscores the impact of extensive atrial remodeling on long-term outcomes, despite an initially favorable response.

However, it is important to note that the CEASE-AF trial used different energy sources for ablation, which may contribute to the differences in outcomes. Therefore, the HARTCAP and CEASE-AF trials are discussed to provide context rather than to imply equivalence or superiority, and differences in outcomes should be interpreted primarily in light of variations in patient populations and procedural strategies rather than epicardial clamp technology.

Safety outcomes were also favorable in patients undergoing hybrid AF ablation using a bipolar irrigated RF clamp, with no significant differences observed between the 1- and 2-stage groups. Importantly, both stroke and mortality rates were 0%. These results are in line with previous studies. In a recent study by Aerts et al,^13^ the early mortality rate for patients undergoing hybrid AF ablation was 1.0%. and 0.1% for early stroke.

In interpreting the observed differences in ICU (2 days vs 1 day; *P* < .001) and total hospital stay (6 days vs 5 days; *P* < .001), it is important to acknowledge that these metrics are strongly influenced by institutional practices rather than procedural complexity alone. ICU duration, discharge policies, and reimbursement structures vary substantially between centers and may therefore confound direct comparison of postoperative recovery between the 1- and 2-stage approaches. As such, the longer ICU and hospital stay observed in the 2-stage group should be viewed with caution and may not necessarily reflect inherent differences in clinical outcomes.

### Redo procedures

Interestingly, several patients in our cohort underwent redo procedures during follow-up. The presenting arrhythmias varied, but, in all cases, persistent isolation of the epicardial lines—both the PVs and the box—was confirmed, suggesting that recurrences were likely driven by arrhythmogenic substrate beyond the PVs and posterior box. Additional ablations were primarily performed at the level of the mitral isthmus and the base of the LAA or involved antral modification of existing endocardial lesions.

### Limitations

Although this is the first study that is equipped to evaluate the EP findings of the hybrid approach, using a biparietal irrigated RF clamp, upon endocardial validation, providing unique insights, it is limited by its nonrandomized design, retrospective character, and relatively small sample size. Consequently, the absence of statistical significance for some outcomes does not completely rule out the potential presence of clinically relevant differences between groups. In addition, the numerical difference in epicardial lesion efficacy between groups, although not statistically significant, may reflect intercenter variability and potential learning curve effects, which could influence reproducibility in broader real-world practice. Furthermore, arrhythmia recurrence was assessed through standard institutional follow-up protocols (including the use of electrocardiography, Holter, and/or ILR), which may underestimate asymptomatic episodes. Nevertheless, this is a reflection of real-world practice. In addition, institutional practices regarding patient selection and hospitalization may differ, which cannot be accounted for. Finally, the impact of adjunctive ablations (eg, mitral isthmus, intercaval lines) was not analyzed.

## Conclusion

Hybrid AF ablation using a biparietal irrigated RF clamp achieved a high percentage of PVs and LAPW isolation, with comparable lesion completeness and safety outcomes in both 1- and 2-stage procedures. Endocardial validation proved valuable for confirming lesion integrity and guiding targeted substrate modification. In the small subset of patients undergoing redo procedures, epicardial lesions of the PVs and LAPW appeared intact; however, given that only limited follow-up data were available, long-term lesion durability could not be conclusively established.
